# hsa_circ_0095812 accelerates periodontitis progression by adsorbing miR-485-3p-mediated THBS1 expression

**DOI:** 10.1016/j.clinsp.2025.100631

**Published:** 2025-04-11

**Authors:** XiaoTing Xie, RuiTing Li, FangLin Mi

**Affiliations:** North Sichuan Medical College, Nanchong City, Sichuan Province, PR China

**Keywords:** Periodontitis, circLRRC4C, miR-485-3p, THBS1, Periodontal Ligament Cells

## Abstract

**Objective:**

To explore the role of hsa_circ_0095812 (circLRRC4C) in periodontitis and its mechanism with miR-485-3p and Thrombospondin-1 (THBS1).

**Methods:**

Periodontal tissues were collected from periodontitis patients. Periodontal Ligament Cells (PDLCs) were stimulated with Lipopolysaccharide (LPS) and transfected. Cell viability, inflammation, apoptosis, and pyroptosis were analyzed. A mouse model of periodontitis was constructed and injected with a lentiviral plasmid vector targeting circLRRC4C. Immunohistochemistry was performed on the periodontal tissue of model mice. The relevant expression level of genes was measured via real-time reverse transcriptase-polymerase chain reaction or Western blot. The relationship between circLRRC4C and THBS1 with miR-485-3p was analyzed.

**Results:**

CircLRRC4C was highly expressed in periodontitis tissues of patients and LPS-treated PDLCs. Downregulating circLRRC4C attenuated LPS-induced PDLC inflammation, apoptosis and pyroptosis and recovered cellular viability. CircLRRC4C acted as a sponge for miR-485-3p. CircLRRC4C affected LPS-induced PDLC apoptosis, pyroptosis and inflammation by regulating miR-485-3p. THBS1 was the target gene of miR-485-3p. Inhibition of THBS1 effectively improved LPS-induced periodontitis. CircLRRC4C aggravated LPS-induced PDLC apoptosis, pyroptosis and inflammation by regulating the miR-485-3p/THBS1 axis. Suppressing circLRRC4C effectively improved periodontitis in mice.

**Conclusion:**

CircLRRC4C induces periodontitis progression by adsorbing miR-485-3p-mediated THBS1 expression.

## Highlights

• circLRRC4C is highly expressed in periodontitis tissues.
• circLRRC4C acts as a sponge for miR-485-3p.
• •THBS1 is a target gene of miR-485-3p.
• Knockdown of circLRRC4C ameliorates periodontitis in mice.

## Introduction

Periodontitis, a long-term, complex inflammatory condition, involves the buildup of dental plaque, known as dental biofilm/biofilm, and is marked by the gradual deterioration of the teeth-supporting apparatus, encompassing the periodontal ligament and alveolar bone.^1^ A complex dynamic interaction between specific bacterial pathogens, destructive host immune responses, and cigarette smoke contributes to the disease.^2^ It is now thought that periodontitis advances through recurrent acute episodes. Throughout their lives, individuals suffering from periodontitis display a pattern of rapid destruction at specific locations for brief durations, succeeded by extended remission periods.^3^ Current treatments include mainly non-surgical treatments, such as scaling and root planning, and surgical treatments, including flap surgery and tissue regeneration. These traditional methods are effective but have limited efficacy, especially in severe and recurrent cases of periodontitis.^3^ Therefore, the search for new therapeutic tools may not only improve treatment outcomes, but may also improve patients' quality of life, especially in preventing disease recurrence and promoting tissue regeneration.

Genetic and epigenetic factors, along with periodontal biofilms, have been identified as factors affecting periodontitis progression.^4^ Epigenetic factors, notably noncoding RNAs (ncRNAs), have also been described as significant players in periodontitis pathogenesis besides gene expression profiles in diseased periodontal tissues.^5^ Circular RNAs (circRNAs), a type of ncRNAs, emerge from the covalent linkage at the end of an individual RNA molecule, a result of a back-splicing expression variation. Research on periodontal tissue and diseases has focused on the variance in circRNA expression during the osteogenic differentiation of human Periodontal Ligament Stem Cells (PDSLCs).^6^^,^^7^ In addition to regulating PDSLC apoptosis and proliferation, circRNAs also exert influence over migration, inflammation, and osteoblast differentiation.^8^, ^9^, ^10^, ^11^ There has been growing interest in the role played by hsa_circ_0095812 (circLRRC4C), a newly discovered circRNA in the progression of periodontitis. miR-485-3p was a predicted target of hsa_circ_0095812 in this study. In fact, miR-485-3p is a differentially expressed miRNA in exosomes of PDSLCs due to mechanical force.^12^ miR-485-3p has been identified to be involved in osteogenic differentiation of PDSLCs.^13^ There is evidence that tooth germ Extracellular Matrix (ECM) components, including Thrombospondin-1 (THBS1), contribute to the remodeling of the ECM.^14^ However, the potential interactions among circLRRC4C, miR-485-3p, and THBS1, and their implications for periodontitis remain to be elucidated.

As part of this study, the authors examined the effects of the circLRRC4C/miR-485-3p/THBS1 axis on periodontitis, with the goal of providing a clinical reference for preventing and managing periodontitis.

## Materials and methods

### Clinical sample acquisition

Periodontal tissues were obtained from March 2017 to May 2018 from patients who underwent periodontal surgery in North Sichuan Medical College. To reduce the impact of varying mechanical forces across different root segments, the authors specifically collected PDLCs from the distal aspect of the middle third (mid-root portion) of the third molar's long axis. This location was chosen because it provides a more accessible surgical field during crown lengthening procedures and minimizes potential sampling bias compared with the apical or cervical thirds. All included third molars were fully or predominantly erupted and were not in infraocclusion (i.e., they were not significantly below the occlusal plane), thus further reducing confounding effects from abnormal occlusal forces. For each of the 17 healthy volunteers, one-third of the periodontal ligament in the aforementioned region was meticulously harvested under magnification to ensure both cellular integrity and consistency across samples. During this process, the authors took care to avoid contamination from adjacent tissues, and we maintained consistent sampling methods (e.g., orientation, depth, and amount of harvested tissue) to minimize variability.

Tissue samples from inflammatory lesions were collected from 28 patients diagnosed with periodontitis who received periodontal flap surgery at North Sichuan Medical College. In cases of periodontitis, patients underwent periodontal flap surgery once the probing depth exceeded 5 mm for an extended duration of 3 months following subgingival debridement. Every participant in this research was free from any systemic disease history and hadn't utilized antibiotics or hormones in the previous three months. Participants were specifically selected based on their non-smoking status and absence of alcohol consumption for at least one week prior to tissue collection. Furthermore, none of the participants had taken non-steroidal anti-inflammatory drugs within 72 hours of the procedure. The periodontal samples were quickly preserved at -80°C. The Ethics Committee of North Sichuan Medical College (nº 20160731CB) sanctioned this study, and every patient provided their written informed consent. To address potential demographic influences, the authors have included a table (Supplementary Table 1) summarizing the age range, mean age, and sex distribution (male/female ratio) for both the healthy and periodontitis groups.

To ensure the statistical validity of the present findings, a sample size calculation was performed prior to the initiation of the study. The calculation was based on detecting a significant difference in the inflammatory markers between healthy and periodontitis-affected periodontal tissues with a power of 80% and an alpha level of 0.05. Preliminary data from a pilot study indicated a standard deviation of these markers in both groups. Using these parameters, it was estimated that a minimum of 15 patients per group would be necessary to detect a clinically relevant difference with the desired power. However, to accommodate potential data loss and to enhance the robustness of the findings, the authors increased the sample size to 17 patients for the group providing healthy tissues and 28 patients for the group with periodontitis. This decision was further supported by similar studies in periodontal research, which commonly employ sample sizes within this range to detect significant differences in periodontal health indicators.

### Cell culture

As mentioned above, PDLCs were isolated from one-third of the periodontal ligament of the third molar of healthy volunteers and digested with trypsin.^15^ Cells were cultured in Dulbecco's Modification of Eagle's Medium (Gibco, USA) containing 10 % fetal bovine serum (Gibco), 100 U/mL penicillin, and 100 μg/mL streptomycin and placed in a humidified incubator at 37 °C, 5 % CO_2_. Cell passaging was performed when the cell confluence reached 80 %, and the third generation of PDLCs was collected for the following experiments. To mimic *in vitro* periodontitis injury, PDLCs were treated with 10 μg/mL Porphyromonas gingivalis Lipopolysaccharide (LPS; Sigma, USA) for 24 h as previously described.^16^

### Actinomycin D and RNase R treatments

To examine the ring structure of circLRRC4C, PDLCs were treated with actinomycin D (A1410, Merck, Germany) and RNase R (RNR07250, Epicenter, USA). Total RNA was isolated using TRizol reagent (Thermo Fisher Scientific), and RNA was incubated for 60 min at 37 °C with RNase R. Actinomycin D (2 mg/mL) or the same concentration of dimethyl sulfoxide (D2650, Merck, Germany) was added to the culture medium and cells were cultured for 4 h, 8 h, 12 h, and 24 h. After treating the cells with RNase R or Actinomycin D, the cells were collected and subjected to real-time Reverse Transcriptase-Polymerase Chain Reaction (RT-qPCR) to detect circLRRC4C and Glyceraldehyde-3-Phosphate Dehydrogenase (GAPDH).

### RT-qPCR

Total RNA extraction was performed utilizing TRizol reagent (Thermo Fisher Scientific). Concerning circRNA and mRNA, the One-Step qRT-PCR kit (D7268M, Beyotime, China) was utilized for reverse transcription and fluorescence quantitative PCR. Regarding miRNA, the RT-qPCR process utilized the miRNA first Strand cDNA Synthesis Kit (MR101, vazyme, China) along with the miRNA Universal SYBR® qPCR Master Mix (MQ101, vazyme). Gene expression levels were measured using the 2^-ΔΔCt^ method through the PCR detection system (CFX96, Bio-rad, USA). GAPDH served as the benchmark gene for circRNA and mRNA. U6 served as a benchmark gene for miRNA. Table 1 contains a detailed listing of the primers.

**Table 1:** Primers.

Genes	Sequences (5′–3′)
circLRRC4C	Forward: 5′-TGACTGACAATCTCTGGGCT -3′
	Reverse: 5′-CACCTGCTGCTACTCTGTCT-3′
miR-485-3p	Forward: 5′-GCGTCATACACGGCTCTC -3′
	Reverse: 5′-TGGTGTCGTGGAGTCG-3′
THBS1	Forward: 5′-AGGACAACTGCAGACTCGTG-3′
	Reverse: 5′-AGAAAGGCCCGAGTATCCCT-3′
U6	Forward: 5′-CTCGCTTCGGCAGCACA-3′
	Reverse: 5′-AACGCTTCACGAATTTGCGT-3′
GAPDH	Forward: 5′-CACCCACTCCTCCACCTTTG-3′
	Reverse: 5′-CCACCACCCTGTTGCTGTAG-3′

THBS1, Thrombospondin-1; GAPDH, Glyceraldehyde-3-Phosphate Dehydrogenase.

### Cell transfection

Small interfering RNAs targeting circLRRC4C and THBS1 and small interfering RNA negative controls (si-circLRRC4C, si-THBS1 and si-NC), overexpression plasmids targeting circLRRC4C and negative controls (pcDNA 3.1 and pcDNA 3.1-circLRRC4C), miR-485-3p mimic/inhibitor and mimic/inhibitor NC were designed and supplied by GenePharma (Shanghai, China). The above plasmids or oligonucleotides were transfected into LPS-treated PDLCs using Lipofectamine 3000 (Invitrogen). After 48h of transfection, transfection efficiency was checked using RT-qPCR or western blot.

### 3-(4,5-dimethylthiazol-2-yl)-2,5-diphenyltetrazolium bromide (MTT) method

PDLCs were introduced into 96-well plates, each containing 3×10^4^ cells. Each well received MTT (5 mg/mL, Sigma) for four hours, followed by the addition of dimethyl sulfoxide (200 μL, Sigma) to dissolve the crystals. Ultimately, a microplate reader (Molecular Devices) identified the absorbance at 570 nm.

### Enzyme-linked immunosorbent assay (ELISA)

Supernatants of PDLCs were collected. Interleukin (IL-) 1β, IL-6 and Tumor Necrosis Factor (TNF)-α, and IL-18 in the supernatant were measured using an ELISA kit (Boster, Wuhan, China). The absorbance at 450 nm was read using a microplate reader (Molecular Devices).

### Flow cytometry

To determine apoptosis, PDLCs were collected and washed with cold PBS. The treated PDLCs were fixed with 70% ethanol and stained with V-Fluorescein isothiocyanate and propidium iodide. Then, a CytoFLEX flow cytometer (Beckman Coulter, USA) was utilized to measure the apoptosis rate.

To determine cellular pyroptosis, active caspase-1 was measured using the FAM-FLICA In Vitro Caspase-1 Assay Kit (ImmunoChemistry, USA). PDLCs underwent a 60 min incubation with FAM-YVAD-FMK at 37 °C in darkness. Subsequently, the cells underwent a staining process using 1 μM SYTOX® Blue stain, a luminescent nucleic acid dye designed to only pierce through damaged cell membranes (Molecular Probes, Eugene, OR, USA), for an extra 10 min at ambient temperature to observe the formation of membrane pores. Post-staining, the cells underwent flow cytometry analysis (Beckman Coulter), identifying cells with double-positive FAM YVAD-FMK and SYTOX® Blue staining as pyroptotic.

### Western blot

PDLCs and periodontal tissues were homogenized in high-efficiency radioimmunoprecipitation assay buffer (Solarbio) supplemented with 1% complete protease inhibitor mixture (Sigma-Aldrich). Protein concentration was determined using the Bicinchoninic Acid Protein Assay Kit (Thermo Fisher Scientific). Protein samples (30 μg) were separated by 12 % sodium dodecyl sulfate-polyacrylamide gel electrophoresis and then electroblotted onto polyvinylidene difluoride membranes. After sealing, the membrane was blotted with p-p65 (3033, Cell Signaling Technology), cleaved caspase-3 (9664, Cell Signaling Technology), p65 (8242, Cell Signaling Technology), NOD-like receptor protein-3 (NLRP3; ab214185, Abcam), cleaved caspase-1 (4199, Cell Signaling Technology) at 4°C overnight. The membrane was then washed and incubated with a secondary antibody (1:10000; ZSGB-BIO, Beijing, China) for 1 hour at room temperature. A chemiluminescence kit (Applygen, Beijing, China) was used to detect protein strips. Intensity levels were quantified using ImageJ software.

### Dual-luciferase reporter assay

Fragments of circLRRC4C/THBS1 with either wild-type or mutant miR-485-3p binding sites were inserted into pGL3 vectors (Promega, USA) to create either wild-type (circLRRC4C-WT, THBS1-WT) or mutant luciferase reporter vectors (circLRRC4C-MUT, THBS1-MUT). The aforementioned vectors, along with miR-485-3p mimic or mimic NC, were transfected into PDLC utilizing Lipofectamine 3000 (Invitrogen). Luciferase activity was identified 48 hours post-transfection through the Dual-Luciferase Reporter Gene Assay Kit (Promega).

### RNA immunoprecipitation (RIP) experiments

EZ-Magna RIP Kit (Millipore, USA) was employed for RIP analysis. After lysis of PDLCs, cell lysates were reacted with magnetic beads conjugated with anti-Ago2 or anti-immunoglobulin G. Finally, the abundance of RNA was checked using RT-qPCR.

### Mouse periodontitis model

Twenty BALB/c mice (male, 5‒6 weeks old) were purchased from Hunan SJA Laboratory Animal Co., Ltd. The mice were housed in an environment with 24°±2°C and a humidity of 50 %‒60 %, and had free access to basal feed and drinking water. After one week of acclimatization, the mice were randomly divided into four groups: The control group, Model group, sh-NC group, and sh-circLRRC4C group. The Model mice were modeled for periodontitis by Porphyromonas gingivalis (ATCC: 33277) infection.^17^ Porphyromonas gingivalis was chosen to induce periodontitis in BALB/c mice due to its status as a keystone pathogen with numerous virulence factors like lipopolysaccharides, gingipains, and fimbriae, which facilitate colonization, disrupt host immune responses, and degrade tissue structures, making it highly relevant for studying periodontal disease mechanisms and therapeutic interventions. To determine the biological function of circLRRC4C, 200 μL of lentiviral plasmid vectors targeting sh-NC and sh-circLRRC4C (2×10^8^ TU/mL, GenePharma) were injected into mice 1-week prior to Porphyromonas gingivalis infection. Mice in the Control group did not undergo Porphyromonas gingivalis infection. After 3-weeks of infection, mice were executed, and periodontal tissues were collected for subsequent analysis of histopathological staining or RNA and protein extraction. Animal experiments followed ARRIVE guidelines and were approved by the North Sichuan Medical College Animal Care Committee (nº 20170411CB).

### Immunohistochemistry

Tissues from the periodontal were preserved in 4 % paraformaldehyde for a day and then decalcified using a 10 % ethylenediaminetetraacetic acid solution for five weeks at ambient temperature. Every sample was encased in paraffin blocks, and sections measuring 4 μm were readied. Sections were subsequently deparaffinized and rehydrated. Sections were incubated with anti-IL-1β (16806-1-AP, Proteintech), cleaved caspase-1 (4199, Cell Signaling Technology), and NLRP3 (ab214185, Abcam) antibodies overnight at 4°C, followed by incubation with biotinylated goat immunoglobulin G for 30 min. Immunoreactivity was observed using diaminobenzidine (Beyotime, Shanghai, China). Images were captured using an Olympus BX optical microscope (Olympus Optical, Tokyo, Japan) with a camera attachment.

### Data analysis

Statistical evaluation was conducted utilizing the SPSS software (IBM, NY, USA). The data were presented as mean ± standard deviation. The Student's *t*-test was utilized to examine the difference between the two groups. A unidirectional variance analysis was utilized in the testing of multiple groups. In cases where analysis of variance showed significance, additional least significant difference post hoc analyses were conducted; p < 0.05 was considered to indicate a statistical difference.

## Results

### circLRRC4C is highly expressed in periodontitis tissues

**Fig. 1:**
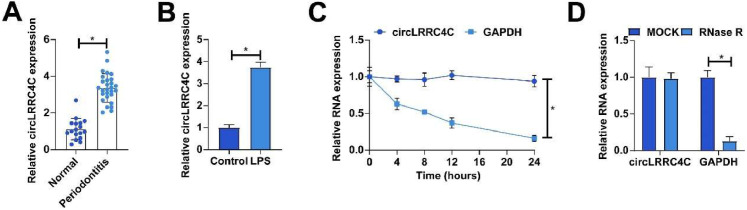
circLRRC4C is highly expressed in periodontitis tissues. (A) RT-qPCR to detect circLRRC4C expression in periodontal tissues of periodontitis patients and healthy subjects; (B) RT-qPCR to detect circLRRC4C expression in LPS-treated PDLCs; (C‒D) Actinomycin D and RNase R assays to examine circLRRC4C's ring structure. Data are expressed as mean ± SD (n = 3). * p < 0.05.

A previous high-throughput sequencing showed that hsa_circ_0095812 (circLRRCRC) was abnormally highly expressed in periodontitis.^18^ This result was further confirmed in the present study. RT-qPCR demonstrated that circLRRC4C expression was significantly higher in periodontitis patient tissues than in normal periodontal tissues (Fig. 1A). In addition, increased circLRRC4C expression was also found in LPS-treated PDLCs (Fig. 1B). To determine the results of circLRRC4C, the authors performed actinomycin D and RNase R experiments. The results showed that circLRRC4C treated with the transcriptional inhibitor actinomycin D had a longer half-life compared with the linear mRNA GAPDH, and RNase *R* treatment was unable to degrade circLRRC4C, suggesting that circLRRC4C has a stable ring structure (Fig. 1C‒D).

### Knockdown of circLRRC4C attenuates LPS-induced inflammation, apoptosis, and pyroptosis in PDLCs

**Fig. 2:**
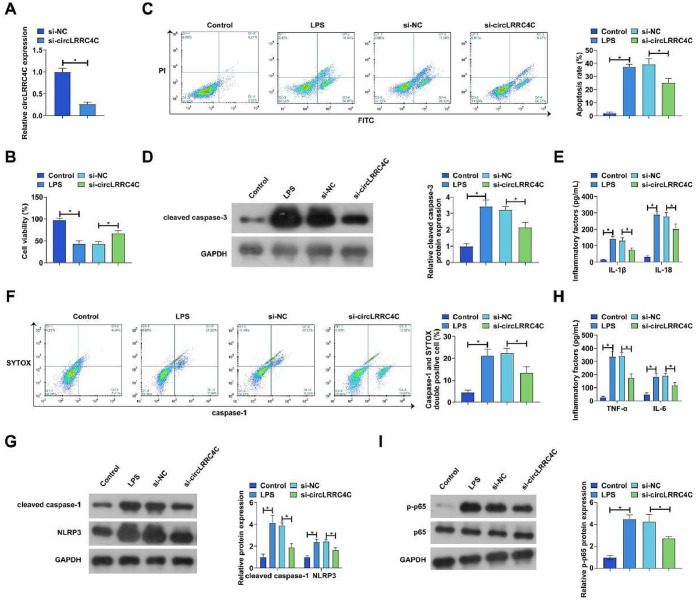
Knockdown of circLRRC4C attenuates LPS-induced PDLC inflammation, apoptosis, and pyroptosis. si-circLRRC4C was transfected into LPS-treated PDLCs. (A) circLRRC4C expression was detected by RT-qPCR; (B) Cell viability was detected by MTT assay; (C) Apoptosis was detected by flow cytometry; (D) Expression of apoptosis marker cleaved caspase-3 was detected by western blot; (E) IL-1β and IL-18 levels in culture medium supernatants were measured by ELISA; (F) Percentage of activated caspase-1 positive cells was measured by flow cytometry; (G) Protein expression of cleaved caspase-1 and NLRP3 was measured by western blot; (H) IL-6 and TNF-α levels in the culture medium were detected by ELISA; (I) Phosphorylation levels of p65 were detected by western blot. Data are expressed as mean ± SD (n = 3). * p < 0.05.

Subsequently, the authors transfected siRNA targeting circLRRC4C into LPS-treated PDLC to explore the biological function of circLRRC4C. si-circLRRC4C significantly reduced circLRRC4C expression in PDLCs (Fig. 2A). PDLC viability was examined by the MTT method. The results showed that the effect of LPS treatment on PDLC viability was ameliorated by knockdown of circLRRC4C (Fig. 2B). Flow cytometry showed that PDLC apoptosis was increased by LPS treatment but was alleviated by knocking down circLRRC4C (Fig. 2C). Subsequently, cleaved caspase-3 was examined by western blot. As shown in Fig. 2D, LPS resulted in increased expression of cleaved caspase-3, while knockdown of circLRRC4C inhibited cleaved caspase-3 expression. The authors then examined the cellular pyroptosis phenomenon. The authors first examined changes in IL-1β and IL-18 by ELISA. Consistent with speculation, LPS treatment resulted in increased levels of IL-1β and IL-18, whereas knockdown of circCRRC4C effectively reduced the levels of these 2 inflammatory factors (Fig. 2E). In addition, LPS treatment promoted an increase in the proportion of positive cells for activated caspase-1, whereas knockdown of circCRRC4C inhibited the number of positive cells for activated caspase-1 (Fig. 2F). Western blot showed that LPS treatment promoted the protein levels of cleaved caspase-1 and NLRP3, but knockdown of circCRRC4C prevented the activation of both proteins (Fig. 2G). Finally, the inflammation of the cells was assessed by ELISA and western blot. The results showed that the levels of inflammatory factors increased and the NF-κB p65 pathway was activated after LPS treatment, whereas knockdown of circCRRC4C significantly suppressed the inflammatory response (Fig. 2H‒I).

### circLRRC4C acts as a sponge for miR-485-3p

**Fig. 3:**
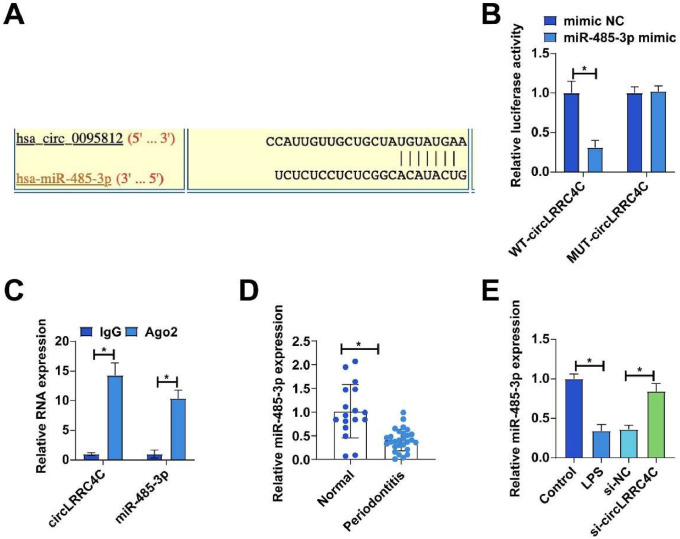
CircLRRC4C acts as a sponge for miR-485-3p. (A) Bioinformatics website https://circinteractome.nia.nih.gov/ to predict the potential binding sites of circLRRC4C and miR-485-3p; (B‒C) Dual luciferase reporter assay and RIP assay to verify the targeting relationship between circLRRC4C and miR-485-3p; (D) RT-qPCR to detect the expression of miR-485-3p in periodontal tissues of periodontitis patients and healthy subjects; (E) RT-qPCR to to detect the effect of si-circLRRC4C on miR-485-3p expression in PDLCs. Data are expressed as mean ± SD (n = 3). * p < 0.05.

Next, the authors explored the downstream miRNAs of circLRRC4C. Potential binding sites for circLRRC4C and miR-485-3p were identified by bioinformatics website prediction (Fig. 3A). The targeting relationship between circLRRC4C and miR-485-3p was subsequently validated by dual luciferase reporter assay and RIP assay. As shown in Fig. 3B, co-transfection of wild-type circLRRC4C with mir-485-3p mimic decreased luciferase activity in PDLCs, but co-transfection of mutant circLRRC4C with miR-485-3p mimic was unable to affect luciferase activity. Moreover, significant enrichment of circLRRC4C and miR-485-3p was found in Ago2 magnetic beads (Fig. 3C). Notably, the authors found abnormally low expression of miR-485-3p in periodontitis tissues (Fig. 3D). Subsequently, the authors examined whether miR-485-3p was regulated by circLRRC4C in PDLC. Consistent with speculation, transfection of si-circLRRC4C effectively increased miR-485-3p expression (Fig. 3E).

### Inhibition of periodontitis by knockdown of circLRRC4C is reversed by downregulating miR-485-3p

**Fig. 4:**
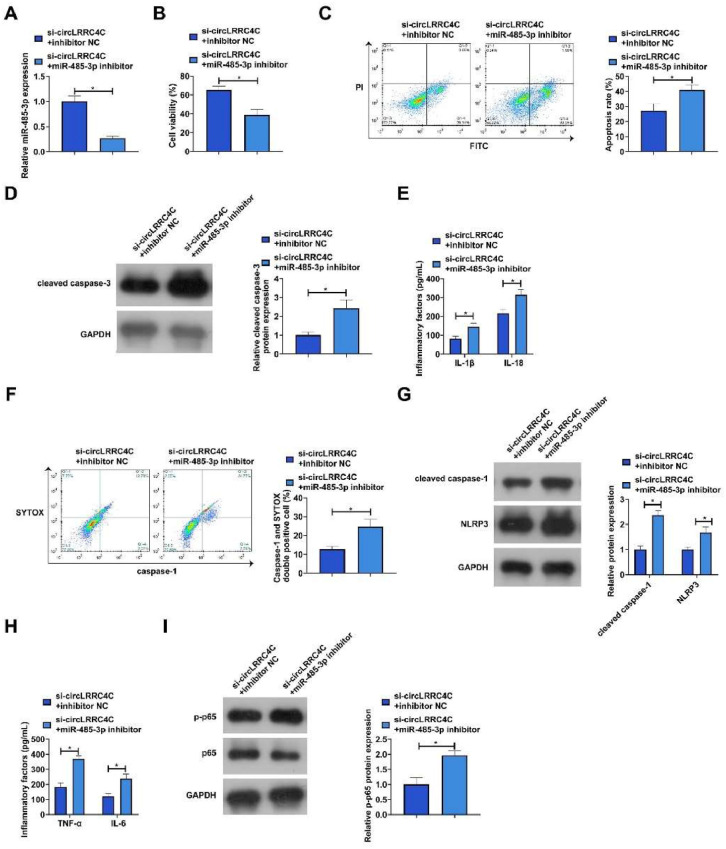
Inhibition of periodontitis by knockdown of circLRRC4C is reversed by downregulation of miR-485-3p. si-circLRRC4C and miR-485-3p inhibitor were co-transfected into LPS-treated PDLCs. (A) miR-485-3p expression was detected by RT-qPCR; (B) Cell viability was detected by MTT assay; (C) Apoptosis was detected by flow cytometry; (D) Expression of apoptosis marker cleaved caspase-3 was detected by western blot; (E) IL-1β and IL-18 levels in culture medium supernatants were measured by ELISA; (F) Percentage of activated caspase-1 positive cells was measured by flow cytometry; (G) Protein expression of cleaved caspase-1 and NLRP3 was measured by western blot; (H) IL-6 and TNF-α levels in the culture medium were detected by ELISA; (I) Phosphorylation levels of p65 were detected by western blot. Data are expressed as mean ± SD (n = 3). * p < 0.05.

Subsequently, the authors explored whether miR-485-3p was involved in circLRRC4C regulation of periodontitis by functional rescue experiments. The authors co-transfected si-circLRRC4C and miR-485-3p inhibitor into LPS-treated PDLCs. RT-qPCR demonstrated that the promotion of miR-485-3p expression by si-circLRRC4C was reversed by miR-485-3p inhibitor (Fig. 4A). In addition, co-transfection with miR-485-3p inhibitor decreased cell viability and promoted apoptosis rate as well as cleaved caspase-3 expression (Fig. 4B‒D). Co-transfection of miR-485-3p inhibitor promoted the levels of IL-1β and IL-18, and increased the proportion of positive cells for activated caspase-1 as well as the protein expression of cleaved caspase-1 and NLRP3 (Fig. 4E‒G). Co-transfection with miR-485-3p inhibitor also resulted in increased levels of inflammatory factors and p65 phosphorylation (Fig. 4H‒I).

### THBS1 is a target gene of miR-485-3p

**Fig. 5:**
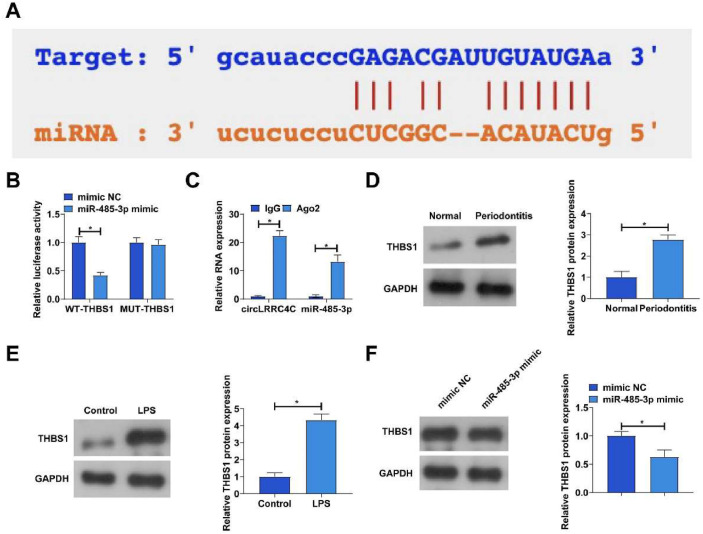
THBS1 is a target gene of miR-485-3p. (A) Bioinformatics website https://circinteractome.nia.nih.gov/ to predict the potential binding sites of THBS1 and miR-485-3p; (B‒C) Dual luciferase reporter assay and RIP assay to verify the targeting relationship between THBS1 and miR-485-3p; (D) Western blot to detect THBS1 expression in periodontal tissues of periodontitis patients and healthy subjects; (E) Western blot to detect THBS1 expression in LPS-treated PDLCs; (F) Western blot to detect the effect of miR-485-3p mimic on THBS1 expression in PDLCs. Data are expressed as mean ± SD (n = 3). * p < 0.05.

Next, the authors predicted the downstream target genes of miR-485-3p by the bioinformatics website. THBS1 was identified as a candidate gene with a potential binding site to miR-485-3p (Fig. 5A). In addition, dual luciferase reporter assay and RIP assay identified the targeting relationship between miR-485-3p and THBS1. Co-transfection of wild-type THBS1 and miR-485-3p mimic reduced luciferase activity, and significant enrichment of THBS1 and miR-485-3p was found in Ago2 magnetic beads (Fig. 5B‒C). Subsequently, the authors examined whether THBS1 was regulated by miR-485-3p in the periodontitis model. As shown in Fig. 5D‒F, THBS1 expression was increased in periodontitis tissues and LPS-treated PDLCS, whereas overexpression of miR-485-3p suppressed THBS1 protein levels in PDLCS.

### Knockdown of THBS1 ameliorates LPS-induced apoptosis, pyroptosis and inflammation in PDLCs

**Fig. 6:**
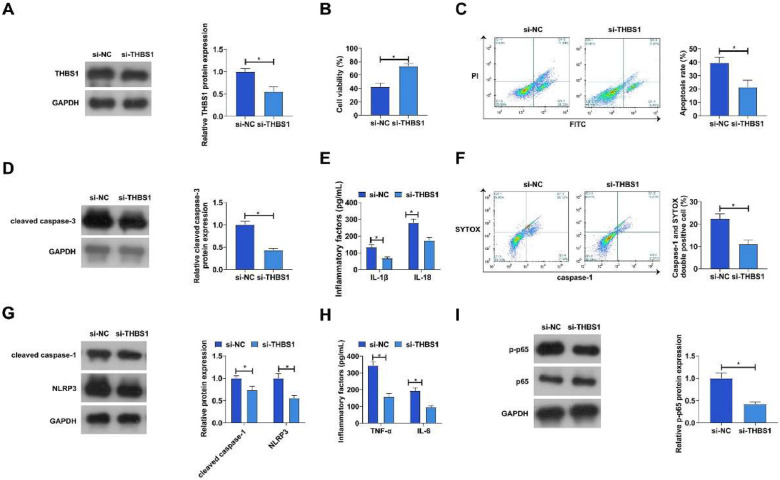
Knockdown of THBS1 ameliorates LPS-induced PDLC apoptosis, pyroptosis, and inflammation. si-THBS1 was transfected into LPS-treated PDLCs. (A) HBS1 expression was detected by Western blot; (B) Cell viability was detected by MTT assay; (C) Apoptosis was detected by flow cytometry; (D) Expression of apoptosis marker cleaved caspase-3 was detected by western blot; (E) IL-1β and IL-18 levels in culture medium supernatants were measured by ELISA; (F) Percentage of activated caspase-1 positive cells was measured by flow cytometry; (G) Protein expression of cleaved caspase-1 and NLRP3 was measured by western blot; (H) IL-6 and TNF-α levels in the culture medium were detected by ELISA; (I) Phosphorylation levels of p65 were detected by western blot. Data are expressed as mean ± SD (n = 3). * p < 0.05.

The authors transfected si-YHBS1 into LPS-treated PDLC to down-regulate THBS1 expression (Fig. 6A). The results showed that knockdown of THBS1 increased cell viability while apoptosis rate and expression of apoptosis-related protein cleaved caspase-3 decreased in PDLCs (Fig. 6B‒D). In addition, down-regulation of THBS1 alleviated PDLC pyroptosis, which was mainly manifested by reduced IL-1β and IL-18 levels, the proportion of positive cells for activated caspase-1, and the expression of pyroptosis-associated proteins cleaved caspase-1 and NLRP3 proteins (Fig. 6E‒G). Downregulation of THBS1 also suppressed TNF-α and IL-6 and limited p65 phosphorylation (Fig. 6H‒I).

### circLRRC4C affects LPS-induced PDLC apoptosis, pyroptosis and inflammation by modulating the miR-485-3p/THBS1 axis

**Fig. 7:**
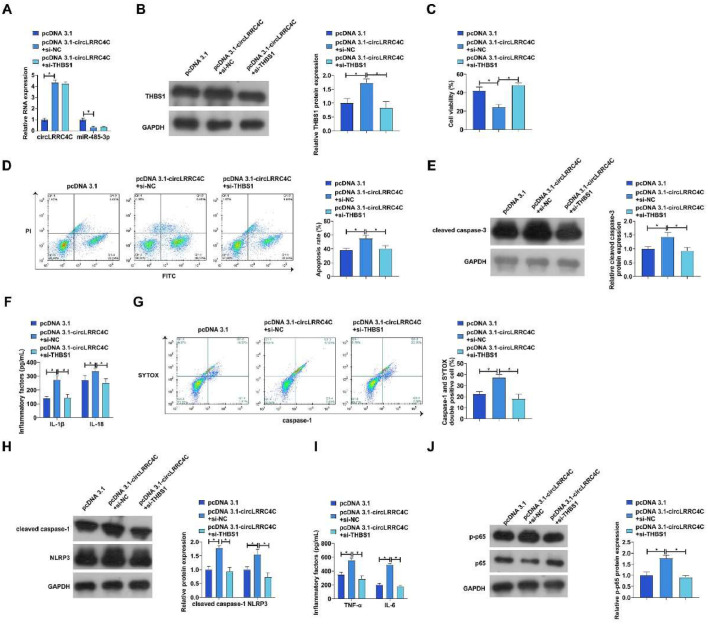
CircLRRC4C affects LPS-induced PDLC apoptosis, pyroptosis and inflammation by regulating the miR-485-3p/THBS1 axis. pcDNA 3.1-circLRRC4C and si-THBS1 were cotransfected into LPS-treated PDLCs. (A) circLRRC4C and miR-485-3p expression was detected by RT-qPCR; (B) THBS1 expression was detected by Western blot; (C) Cell viability was detected by MTT assay; (D) Apoptosis was detected by flow cytometry; (E) Expression of apoptosis marker cleaved caspase-3 was detected by western blot; (F) IL-1β and IL-18 levels in culture medium supernatants were measured by ELISA; (G) Percentage of activated caspase-1 positive cells was measured by flow cytometry; (H) Protein expression of cleaved caspase-1 and NLRP3 was measured by western blot; (I) IL-6 and TNF-α levels in the culture medium were detected by ELISA; (J) Phosphorylation levels of p65 were detected by western blot. Data are expressed as mean ± SD (n = 3). * p < 0.05.

The authors hypothesized that circLRRC4C might exacerbate periodontitis progression by modulating the miR-485-3p/THBS1 axis. To verify this conjecture, the authors co-transfected pcDNA 3.1-circLRRC4C and si-THBS1 into LPS-treated PDLCs. RT-qPCR and western blot demonstrated that pcDNA 3.1-circLRRC4C inhibited miR-485-3p and promoted circLRRC4C and THBS1 expression, while si-THBS1 had no effect on miR-485-3p and circLRRC4C expression but downregulated THBS1 expression (Fig. 7A‒B). Overexpression of circLRRC4C inhibited cell viability and promoted apoptosis rate and protein levels of cleaved caspase-3, but knockdown of THBS1 prevented these changes (Fig. 7C‒E). Overexpression of circLRRC4C upregulated the levels of IL-1β and IL-18, increased the proportion of activated caspase-1-positive cells, and promoted the expression of cleaved caspase-1 and NLRP3, whereas knockdown of THBS1 effectively inhibited the pyroptosis phenomenon in PDLCs (Fig. 7F‒H). In addition, overexpression of circLRRC4C also exacerbated LPS-induced inflammation in PDLC, but knockdown of THBS1 effectively ameliorated inflammation in PDLCs (Fig. 7I‒J)

### Knockdown of circLRRC4C ameliorates periodontitis in mice

**Fig. 8:**
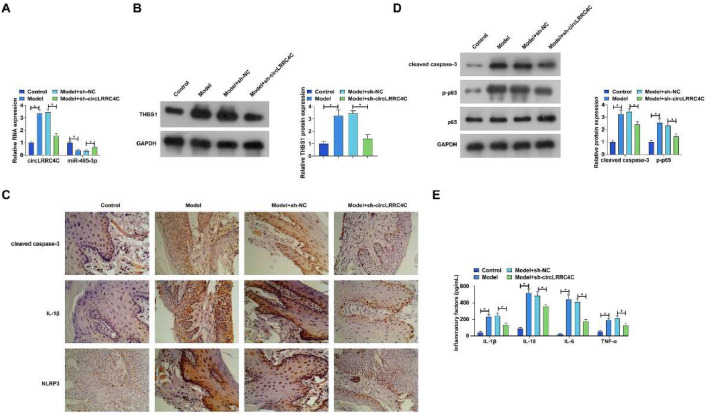
Knockdown of circLRRC4C ameliorates periodontitis in mice. sh-circLRRC4C lentiviral plasmid was injected into Porphyromonas gingivalis-infected mice. (A) circLRRC4C and miR-485-3p were examined by RT-qPCR; (B) THBS1 was assessed by western blot; (C) IL-1β, cleaved caspase-1, and NLRP3 were measured by IHC; (D) TNF-α, IL-1β, IL-18, and IL-6 in periodontal tissues were detected by ELISA; (E) Cleaved caspase-3 and phosphorylated p65 were detected by western blot. Data were expressed as the mean ± SD (n = 5). * p < 0.05.

To further support the *in vitro* results of this study, the authors subsequently performed *in vivo* experiments. Periodontitis was modeled by Porphyromonas gingivalis infection and sh-circLRRC4C lentiviral plasmid was injected to reduce circLRRC4C expression. Changes in the circLRRC4C/miR-485-3p/THBS1 axis were first evaluated by RT-qPCR and western blot. As shown in Fig. 8A‒B, Porphyromonas gingivalis infection promoted the expression of circLRRC4C and THBS1 but suppressed the expression of miR-485-3p, but this effect was reversed by knockdown of circLRRC4C. Immunohistochemistry showed that Porphyromonas gingivalis infection promoted the expression of IL-1β, cleaved caspase-1, and NLRP3, whereas knockdown of circLRRC4C effectively reduced the number of positive cells for these three proteins (Fig. 8C). ELISA results showed that knockdown of circLRRC4C effectively suppressed the expression of IL-1β, IL-1β and NLRP3 due to Porphyromonas gingivalis infection (Fig. 8C). ELISA results showed that knockdown of circLRRC4C effectively inhibited the increase of TNF-α, IL-1β, IL-18 and IL-6 levels caused by Porphyromonas gingivalis infection (Fig. 8D). In addition, western blot data revealed protein changes in the periodontitis model. As shown in Fig. 8E, Porphyromonas gingivalis infection promoted the expression of cleaved caspase-3 and phosphorylated p65, whereas knockdown of circLRRC4C alleviated the changes in these proteins.

## Discussion

LPS was employed to provoke inflammatory states in periodontitis, and models induced by LPS, both *in vitro* and *in vivo*, have been extensively utilized to explore the molecular mechanism of periodontitis development.^19^^,^^20^ circLRRC4C/miR-485-3p/THBS1 axis expression profiles and mechanisms were validated in periodontitis tissues and cells induced by LPS.

Within the reported mechanisms, multiple pathways leading to programmed cell death have been identified as playing a role in periodontitis. Up until now, over ten varieties of programmed cell death pathways have been recognized, with research focusing on their functions in periodontitis. Despite the extensive investigation of apoptosis in periodontitis, little is known about pyroptosis and its relation to apoptosis. It has been documented that pyroptosis is linked to a widespread inflammatory response during periodontitis. It has been shown that pyroptosis in periodontal tissue can trigger inflammation, leading to damage to these tissues. Elevated pyroptosis in periodontitis may trigger the release of active inflammatory agents, thereby intensifying the inflammatory reaction and resulting in an excessively active immune response. This leads to reduced bone growth, increased bone resorption due to elevated receptor activator of nuclear factor-kappa B ligand levels, intensified destruction of periodontal tissue, and hindered regeneration.^21^

The abundance of circRNAs in various tissues and their stability makes them ideal as molecular markers for the diagnosis and treatment of a wide range of diseases.^22^ These observations indicated a greater abundance of circLRRC4C in periodontitis tissues and model cells induced by LPS, hinting at circLRRC4C's potential as an immediate diagnostic indicator for periodontitis going forward. Following multiple validations, it was discovered that reducing circLRRC4C levels significantly lessens inflammation, apoptosis, and pyroptosis in PDLCs under LPS stimulation. In the future, circLRRC4C may serve as an effective molecular target for the diagnosis and treatment of periodontitis.

This research pinpointed miR-485-3p as a key target for circLRRC4C in jointly managing LPS-induced damage in PDLCs. miR-485-3p was identified as having a pivotal function in the realm of human illnesses. As an instance, miR-485-3p has been commonly considered a tumor suppressor.^23^^,^^24^ Moreover, miR-485-3p suppresses cell growth and stimulates the differentiation of neural stem cells by focusing on thyroid hormone receptor-interacting protein 6 expression.^25^ During postmenopausal women with osteoporosis, miR-485-3p expression is downregulated and may contribute to vertebral fractures.^26^ The research identified a decrease in miR-491-5p in both periodontitis tissues and cells. Concurrently, the reduction of miR-485-3p nullified the mitigating impact of circLRRC4C knockdown on damage caused by LPS in PDLCs. The present research verified the joint control of the periodontitis process by circLRRC4C and miR-485-3p.

This research confirmed the specific interaction between THBS1 and miR-485-3p, revealing a significant upregulation of THBS1 in both periodontitis tissues and cells. Thrombospondin, a group of matricellular proteins secreted, acts as a mediator in cellular attachment dynamics and the production of extracellular matrix proteins, triggered by stress and injury.^27^ It has been noted that USF2 knockdown downregulates THBS1 to reduce pyroptosis and further ameliorate sepsis-induced acute kidney injury.^28^ In preeclampsia, THBS1 is upregulated, modulating trophoblast fusion via an inhibition of Cyclic adenosine monophosphate by CD36.^29^ circLRRC4C and miR-485-3p were co-regulators of the THBS1 level, while this axis functioned in tandem to regulate periodontitis progression.

The study concluded that silencing circLRRC4C alleviated LPS-induced PDLC injury by targeting the miR-485-3p/THBS1 axis and was therefore useful for future diagnosis and treatment of periodontitis.

## Consent to publish

Written informed consent for publication was obtained from all participants.

## Data available

Data is available from the corresponding author on request.

## Ethics statement

All procedures performed in this study involving human participants were in accordance with the ethical standards of the institutional and/or national research committee and with the 1964 Helsinki Declaration and its later amendments or comparable ethical standards. All subjects were approved by the North Sichuan Medical College (nº 20160731CB). The animal experiments were complied with the ARRIVE guidelines and performed in accordance with the National Institutes of Health Guide for the Care and Use of Laboratory Animals. The experiments were approved by the Institutional Animal Care and Use Committee of North Sichuan Medical College (nº 20170411CB).

## Funding

Not applicable.

## CRediT authorship contribution statement

**XiaoTing Xie:** Conceptualization, Formal analysis, Investigation, Writing – original draft. **RuiTing Li:** Methodology, Investigation, Data curation. **FangLin Mi:** Methodology, Formal analysis, Data curation, Writing – review & editing.

## Declaration of competing interest

The authors declare no conflict of interest.

## Appendix Supplementary materials

Image, application 1
