# Supplementary material for: hsa_circ_0095812 accelerates periodontitis progression by adsorbing miR-485-3p-mediated THBS1 expression
Source: Clinics (Sao Paulo). 2025 Apr 11;80:100631. doi: 10.1016/j.clinsp.2025.100631 (PMC12018572; doi:10.1016/j.clinsp.2025.100631)
Supplement: Supplementary file 1 [file mmc1.docx]

**CLINICS-D-24-00362_Supplementary Material**

**Supplementary Table 1** Characteristics of healthy volunteers and periodontitis patients

| **Characteristic** | **Healthy volunteers**  **(n = 17)** | **Periodontitis patients**  **(n = 28)** | **p-value** |
| --- | --- | --- | --- |
| Gender |  |  |  |
| Male | 11 | 16 | 0.6156 |
| Female | 6 | 12 |  |
| Age (year) | 45.6 ± 5.3 | 47.2 ± 6.1 | 0.36 |
| BMI (kg/m^2^) | 23.5 ± 2.2 | 24.1 ± 2.6 | 0.4133 |
| Smoking |  |  |  |
| Yes | 8 | 13 | 0.9672 |
| No | 9 | 15 |  |
